# 

**Published:** 1890-10-25

**Authors:** 


[Registered at the General Post Office as a Newspaper for transmission in the United Kingdom and Abroad.]

WITH EXTRA NURSING SUPPLEMENT.

Per Annum, 6s. 6d.; post free.

Monthly Parts, 6d.; post free 8d.

Ditto per Annum, 8s., post free.

### THE HOSPITAL

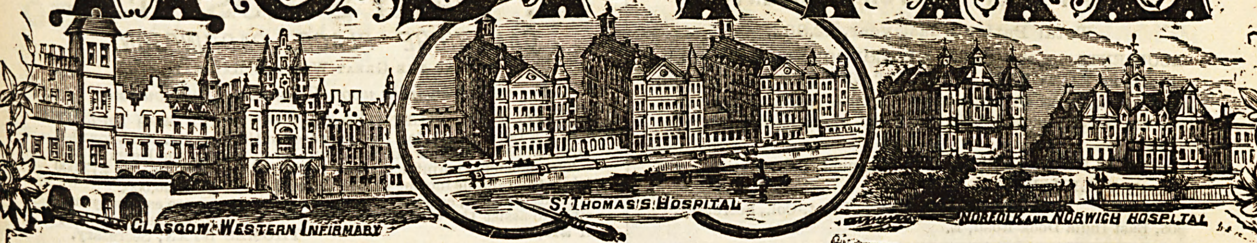

A WEEKLY JOURNAL OF

### Science, Medicine, Nursing and Philanthropy.

SATURDAY, OCTOBER 25th, 1890.

**German Universities and English Students** - 49
**Passing Topics.**—Male Nurses.—II. - 50
**The Doctor's Pulpit.**—The Perfection of Human Happiness - 51
**Everybody's Page** - 52
**Turning Over New Leaves.**—Women and Nerves - 53
**A Department of Public Safety** - 54
**Life Preservation from a Business Point of View** - 54
**Some Curious Patents** - 55
**Notes and News** - 56
**The Story of the Insane from Year to Year** - 57
**Annotations.**—Mixed Committees—Nurses and Balloonists—Students in Pauper Hospitals - 58
**The Practitioner's Mirror and Retrospect:**—
The London Post-Graduate Course at the Paddington Infirmary.—Heart Disease, 59; Pathology - 60
Climatology.—The Climatology of England - 60
New Drugs, Appliances, and Things Medical.—Professor Neugebauer's New Disinfectant - 61
**Ought Women to be on Hospital Committees?** - 61
**General Charities—Scraps and Gleanings** - 62
**The Medical Students' Supplement.**—In the Cause of Medical Students—Notes from the Schools—Men of the Future—Students' Medical Societies, 63; —Scholarships and Prizes—Pass Lists—Athletics—Events for the month - 64

### EXTRA SUPPLEMENT—THE NURSING MIRROR.

**En Passant.**—Watford Nurses—Servants of the Poor—Another Nice Story—Short Items—A Nurses' Hotel—Northern Workhouse Infirmaries - xiii
**Practical Hints for Nurses** - xiv
**Six Months in a Hospital.**—By a "Special Pro." IV. - xiv
**One Reason Why** - xv
**Everybody's Opinion.**—Another Heroine, xv.; The Case of the Older Nurses—Male Nurses - xvi
**Notes and Queries** - xvi
**Appointments** - xvi
**Vacancies** - xvi
**Amusements and Relaxation** - xvi

